# Supplementary material for: Are carbon emissions trading and green financial instruments synergistic? -Comprehensive quantitative research based on content analysis
Source: PLoS One. 2024 Mar 7;19(3):e0298601. doi: 10.1371/journal.pone.0298601 (PMC10919601; doi:10.1371/journal.pone.0298601)
Supplement: S1 Data — (ZIP) [file pone.0298601.s001.zip › Data/地级市Z_F工作报告.zip!/╦─┤¿/├α╤⌠╩╨/└┤╫╘╣1⁄2╓┌║┼í╛┬φ┐╦╩2╛▌═°í┐_2014.docx]

各位代表：

现在，我代表市人民政府，向大会报告工作，请予审查。

2013年工作回顾

2013年，在省委、省政府和市委正确领导下，在市人大、市政协监督支持下，市政府紧紧依靠全市各族人民，深入贯彻党的十八大、十八届二中、三中全会和市委六届五次全会精神，按照科学发展、加快发展工作基调，坚持科技立市、工业兴市，较好完成了市六届人大四次会议确定的主要目标任务，实现了西部经济文化生态强市建设良好开局。预计全年实现地区生产总值1455.12亿元、增长10%，全社会固定资产投资1001亿元、增长7.4%，社会消费品零售总额650亿元、增长13.5%，地方公共财政收入90.48亿元、增长12.5%，城镇居民人均可支配收入23100元、增长11.3%，农民人均纯收入9257元、增长12.7%。

一年来，我们共克时艰，成功应对重大灾害。我市先后遭遇罕见的冬春连旱和“7·9”特大暴雨洪灾袭击，给人民生命财产造成重大损失。全市上下勠力同心、众志成城、抢险救灾，转移安置群众7.9万人，安排应急救灾资金9890万元，补种增播农作物16.5万亩，最大限度抢救群众生命，最大程度减轻灾害损失，打赢了抗旱防汛攻坚战。

一年来，我们锐意进取，科技城建设开启新程。部际协调小组第11次会议成功召开，刘延东副总理出席并提出“五个新”的要求。省政府作出建设100平方公里科技城集中发展区战略决策，首批60个重大项目全面启动。2013中国科技城科技博览会圆满举行，来自15个国家和地区的7480位嘉宾、31万观众参会，参展项目1304项，现场签约金额486.5亿元，公开发表《创新驱动·绵阳共识》，在科技城发展史上奠定了新的里程碑。

一年来，我们敢为人先，创新创业活力迸发。广泛引导各类人才领办创办企业，全年来绵创业人员9190人，认证和新增科技型中小企业1872户，小微企业突破3万户。加快科技成果孵化转化，专利申请4992件、增长38.6%，授权2846件、增长66.2%，两项指标均实现两年翻番。全市研发经费支出占地区生产总值比重6.2%，科技进步综合水平指数64.9%，首次被评为“全国科技进步先进市”。

一年来，我们迎难而上，发展环境显著提升。扎实推进“一评五制三整顿”和“打非治违”专项行动，二环路三期等“7+2”重点项目进展顺利。加快实施煤炭全行业退出工作，建成道路安全防护栏511公里，安全生产形势持续稳定。全面开展平政河综合治理和秸秆禁烧，城区空气质量优良天数达322天，主要流域出境断面水质达标率100%。“天蓝、地绿、水净、人和”的城市新貌日益显现，成功获评“全国十佳最具投资竞争力城市”。

经过大家共同努力，全市综合实力和整体竞争力进一步跃升，科技城知名度和美誉度进一步增强，群众生活水平和幸福指数进一步提高。回顾一年政府工作，我们重点抓了以下几方面。

一、推动经济转型升级，次级突破势头强劲

工业引领作用凸显。“三倍增”计划深入实施，实现规模以上工业增加值593.8亿元、增长10.7%，“2+4”产业实现增加值551.7亿元、增长11.2%。新建四川省北斗卫星导航等4个战略性新兴产业联盟、创新联盟达9个，成功创建国家数字家庭应用示范产业基地。10亿元以上企业达到19户，长虹、九洲、攀长钢、新华4户企业累计实现工业总产值1144亿元。国有出资企业实现工业增加值235亿元、增长16%，非公有制经济增加值占地区生产总值比重达到57.2%。“园区千百亿工程”扎实推进，高新区等5个重点园区营业收入占全市园区的87%，游仙经开区升格省级开发区，江油工业园扩区调位获批。

三次产业结构优化。产业结构由16.3:52.4:31.3调整为16.4:51.4:32.2。国家级杂交水稻制种基地成功授牌，现代农业产业集中连片发展，农林牧渔实现总产值394.97亿元、增长3.6%。川矿等老工业基地调整改造工作有序推进。剑南国际食品城全面建成，经开区万达城市综合体加快建设，建筑业实现产值262.92亿元、增长22.2%。龙门物流园区、毅德商贸城建设进展顺利。金融业健康发展，银行存贷规模3696亿元、增长12.5%，居全省第二位。电商、会展、服务外包等现代服务业发展迅猛，服务业增加值468.55亿元、增长9.1%。小寨子沟升格国家级自然保护区，“5·12”汶川特大地震纪念馆全面开放，北川羌城旅游区晋升5A级景区，全市旅游总收入205.21亿元、增长49.6%。

开放水平日益提高。新设立驻北京、重庆和海外招商分局，积极参与民企入川、央企入川、港澳企业四川行“三大投资促进活动”和财富论坛、世界华商大会、西博会等招商活动，成功引进IBM大数据中心、宝马N20发动机等重大项目。全市国内省外履约项目293个，到位资金473.8亿元、增长9.2%，直接到位外资2.4亿美元、增长14.8%，外贸进出口总额28.1亿美元、增长27%。在境外投资的本土企业达20户、总额9.6亿美元，居全省第二位。外事、台侨、口岸、海关、检验检疫等工作得到加强。

二、突出科技支撑引领，创新驱动成果丰硕

政策支撑更加有力。省政府下发《关于加快推进绵阳科技城建设的指导意见》和《支持绵阳科技城加快建设政策措施》等文件，鼓励绵阳在土地政策、股权激励、成果转化、科技金融等10个方面先行先试。我市研究出台《关于加快科技型中小企业发展的决定》、《关于大力扶持小微企业发展的实施意见》等20余项政策，自上而下的政策体系逐步建立，科技城正成为西部“政策洼地”和“创新高地”。

平台建设务实高效。创新中心A区一期入驻企业和科技服务机构70家，42万平方米的A区二期加快建设，科技城软件产业园顺利挂牌，中科育成中心启动运行。国家大学科技园入驻企业52家，工业技术研究院孵化企业12家、推出产品成果15项。军民融合企业达到261家，其中规模以上78家。长虹、九洲分别被认定为国家首批工业设计中心、国家级技术创新示范企业，全市国家、省级企业技术中心达到53家，建成涵盖15个重点实验室和300余家企业的科研设施共享平台。

创新要素快速集聚。科技金融结合试点扎实开展，两家科技专营银行和科技信贷专营中心作用得到充分发挥，累计发放科技贷款80亿元。与中关村、清华大学等签订战略合作协议，先后引进中国宝安投资、中国风险投资等风投机构，长虹佳华、新晨动力在香港成功上市。“千英百团”聚才计划深入实施，省市设立6000万元人才发展专项资金，一次性拿出1670万元重奖资助优秀人才和创新创业团队，引进各类高层次人才410人，科技城专业人才达21.7万人。

三、统筹城乡协调发展，基础设施显著提升

城区设施不断完善。城市建成区面积107.5平方公里、常住人口118万。绵阳机场通航城市22个，客运总量突破91万人次。成绵乐客专加快建设，绕城高速有序推进，绵西高速即将开工。双碑立交桥、涪江东原大桥、高新区货运通道等缓堵项目完工，新增钢结构人行天桥5座，66个市政基础设施重点项目完成投资32.7亿元。加快实施公共WIFI应用示范工程，安装智能车载系统1237套，推进国家智慧城市试点市、国家信息消费试点市建设。湿地公园、体育公园开工建设，新建城市绿道7.5公里，获得联合国环境规划基金会杰出绿色生态城市奖。

城镇建设步伐加快。绵江安北同城化稳步推进，“大绵阳”半小时交通圈正在形成。游仙魏城、平武南坝等8个全省百镇建设试点镇规划深入实施，梓潼许州、涪城龙门等16个市级试点镇加快建设，江油武都、安县秀水2个5万人以上中心集镇初步建成，转移农村剩余劳动力5.9万人。全市277个集镇建成区面积159.7平方公里、常住人口60.5万人，建成优美示范县城2个、优美示范镇30个，城镇化率45.3%。

乡村面貌日新月异。农村基础设施显著改善，硬化乡村道路2394公里，综合整治农村土地66.1万亩。新增农田有效灌溉面积9.1万亩，武都水库建成蓄水。开展农村土地承包经营权确权登记乡镇85个，涉及农户42万户、耕地155.4万亩。积极推进天然林保护工程，巩固退耕还林成果，深化集体林权制度改革，流转林地22.1万亩。农民增收行政首长负责制全面落实，新增农民专合社470个，注册家庭农场100家。第二轮省级新农村示范县建设全面启动，建成聚居点181个、新农村综合体16个。

四、力促民生持续改善，社会事业全面发展

民生工程深入实施。“十大民生工程”投入资金100.4亿元，完成省市民生项目189个。最低工资标准提高20%，城乡最低生活保障标准上调15%。压缩行政经费提高环卫绿化工人待遇，补贴1000万元支持空调公交车执行1元票价。办理便民自行车IC卡1.6万张，200余万人次使用公共自行车。新增城镇就业4.6万人，城镇登记失业率控制在3.9%以内。开工保障性住房和改造棚户区15244套、竣工13323套，发放廉租住房补贴7395户、共计734万元。“7·9”洪灾灾区恢复重建总体规划全面实施，累计安排救灾重建资金2.5亿元。北川、平武扶贫开发深入推进，全市减少贫困人口10.1万人。投入财政资金4020万元，扎实开展对口支援阿坝州壤塘县、红原县和援建雅安市雨城区工作。

公共服务均衡发展。学前教育三年行动计划深入实施，新（扩)建公办幼儿园28所。素质教育不断推进，高考连续13年全省“双第一”。新增高校1所，西科大成为省部共建高校、取得博士授位权资格。医药卫生体制改革走在全省前列，新农合实现全覆盖，“十二五”国家重大传染病综合防治示范区项目加快实施，全国中医药先进市顺利验收，7家新增三级医院通过评审。食品药品监管机构改革全面完成，查处食品药品违法案件561起。公益性文化事业单位改革稳妥推进，城乡公共文化服务体系逐步健全，被评为全国第二批文化和科技融合示范基地。文艺作品《两个人的车站》获第十届中国艺术节群星奖，广播节目《七色花》获全国少儿节目精品奖。人口素质明显提高，自然增长率控制在1.96‰以内。广播电视、档案、信息化、老龄、气象、水文、地方志、红十字、残疾人、妇女儿童、防震减灾等工作有力推进。

社会管理全面提升。绵阳志愿服务总队被中国志愿服务联合会聘为首批理事单位，全国城市文明程度指数测评在50个文明城市中较2012年上升21位。网络理政深入开展，荣获全国首批网民留言办理工作示范市称号。领导干部下访和包案化解信访积案制度全面推行，重大决策社会稳定风险评估机制逐步健全。平安建设活动积极开展，群众满意度全省第一。市公安局获公安部“4·20”芦山地震抗震救灾嘉奖，市拘留所荣立公安部集体一等功。“六五”普法扎实开展，“绵阳司法”获评全国十大司法行政微博。城市管理水平显著提升，首批设置完成24个便民服务摊区。安全社区和企业安全生产标准化建设不断强化。国防教育和国防动员持续深化，后备力量建设切实加强。统计、工商、税务、邮政、住房公积金、国安、民族、宗教、保密等工作积极推进。

一年来，我们认真落实中央、省、市改进作风各项规定，扎实开展学风文风会风、“三公”经费、节庆论坛、楼堂馆所、会员卡预付卡等五项集中整治行动，市本级压缩“三公”经费1370万元、较年初预算下降10%。我们深入开展政务调研，大力推进政务公开，顺利完成市级部门行政权力目录编制，砍减市级行政审批事项133项，政务服务效能显著提升，涌现了兰辉、张勇等一批先进典型。我们着力加大行政监察和审计监督力度，严格执行重大事项向市人大及其常委会报告、向市政协通报制度，189件人大代表建议、414件政协提案全部按时办结。

各位代表，过去的一年，面对错综复杂的国内外形势和经济下行压力,全市上下锐意进取、攻坚克难，筑牢了多点多极支撑的坚实基础，形成了城乡统筹发展的良好格局，开启了创新引领未来的崭新篇章。这些成绩的取得，是省委、省政府亲切关怀的结果，是市委正确领导，市人大、市政协监督支持的结果，是全市广大干部群众凝心聚力、团结奋斗的结果。在此，我代表市人民政府，向全市各族人民，向驻绵解放军和武警部队官兵、政法干警，向各民主党派、工商联、无党派人士、各人民团体，向所有关心、支持和参与绵阳改革发展的各级领导、各界朋友，表示衷心的感谢，并致以崇高的敬意！

在肯定成绩的同时，我们也清醒地认识到，全市经济社会发展也面临一些不容忽视的困难和问题，主要表现在：受国家宏观政策调整、我市自然灾害频发等因素影响，地区生产总值、规模以上工业增加值、社会消费品零售总额、城镇居民人均可支配收入、农民人均纯收入等5项指标未能完成目标任务；县域发展不平衡，经济质量和效益亟需进一步提高；社会治理水平有待提升，化解社会矛盾、救助弱势群体等任务依然繁重；一些政府部门和工作人员服务意识、责任意识、为民意识、法纪意识不强，在盘江大桥垮塌等事件中存在渎职失职行为；个别地方盲目追求GDP增速，政绩观出现偏差，工业增加值等统计数据失真，损害了政府公信力。在今后工作中，我们将认真总结经验、深刻汲取教训，坚持底线思维、强化问题导向，采取有力措施切实加以解决。

2014年工作安排

今年是在新的起点上全面深化改革的开局之年，是贯彻实施省委、省政府确定的“三大发展战略”，为率先实现次级突破奠定坚实基础的至关重要的一年，是建设西部经济文化生态强市、实现科技城建设突破性发展和实施“十二五”规划的关键之年。实施新一轮综合改革的战略机遇，加快推进西部大开发的历史机遇，国家规划建设丝绸之路经济带和长江流域经济带的新兴机遇，省内首条城际高铁成绵乐客专即将通车的现实机遇，以及科技城建设的独特机遇等交相叠加，绵阳正处于跨越提升、大有可为的关键时期。做好今年工作，意义重大。

今年工作的总体要求：全面贯彻落实党的十八大、十八届二中、三中全会、中央经济工作会议，省委十届三次全会、省委经济工作暨城镇化工作会议以及市委六届六次全会精神，坚持科学发展、加快发展工作指导思想，把握稳中求进、改革创新工作基调，以科技城集中发展区建设和战略性新兴产业为主要抓手，推进科技城建设取得更大突破，围绕建设中国西部经济文化生态强市，继续实施“两化”互动和城乡统筹、创新驱动、开放合作、文化引领、民生优先、绿色低碳“六大发展战略”，促进全市经济持续健康发展、社会和谐稳定。

今年经济社会发展的主要预期目标：地区生产总值增长10%，规模以上工业增加值增长11.5%，全社会固定资产投资增长5%，社会消费品零售总额增长13%，地方公共财政收入增长11%，城镇居民人均可支配收入增长10%，农民人均纯收入增长12.5%，居民消费价格涨幅控制在3.5%左右，城镇登记失业率低于4.3%，人口自然增长率控制在3‰以内，完成省政府下达节能减排任务。

需要说明的是，省委经济工作会议指出，要正确看待经济增速问题，不能盲目追求高增长，也不能把“换挡”变成“失速”，有条件地区可以乘势而进，发展得快一点。在此基础上，我们认真研究、充分论证，把地区生产总值增速确定为10%，这个目标在经济下行压力加大情况下已经是一个较快速度，既考虑了调整结构的现实需要，也考虑了经济运行的实际困难，是一个必须通过艰苦努力才能实现的目标。

今年，我们将重点抓好以下八个方面的工作。

一、加快改革步伐，在推动开放合作上力求新突破

坚持正确、准确、有序、协调推进改革，充分发挥市场在资源配置中的决定性作用，不断增强发展的内驱力。

完善现代市场体系。建立覆盖全市的征信系统，加强市场主体准入、合同履行、产品质量等领域信用信息监管。推进长虹等国有企业改革，健全企业法人治理结构。完善事业单位分类改革政策，激发事业单位活力。深化财税改革，坚持“举债有范、量入为出”，控制地方债务风险。规范征地程序，完善被征地农民多元保障机制。加强金融管理创新，推进商业银行和农村信用社改革，鼓励科技型企业到“新三板”挂牌融资。深化教育、文化、卫生等行业改革，鼓励社会力量兴办公益事业。

壮大非公有制经济。坚持“两个毫不动摇”方针，统筹发展各种所有制经济，探索发展混合所有制经济的有效路径。按照权利平等、机会平等、规则平等，废除对非公有制经济各种不合理规定，切实消除隐性壁垒，让一切劳动、知识、技术、管理、资本的活力竞相迸发。建立非公有制企业贷款风险补偿金，引导东材科技、富临集团等企业转型升级，支持兴事发、六合锻造等企业上市融资。力争非公有制经济增加值占地区生产总值比重57.5%以上，民间投资占全社会投资比重54.5%以上。

扩大开放合作领域。充分发挥7个驻外招商分局作用，加强成都经济区“8+3”区域合作，深度融入成渝经济区，深化与泛珠三角、长三角等区域联系。提升与港澳台地区合作水平，扩大与欧美、日韩等国家和“东盟新四国”等新兴市场经贸交流。积极参加西博会、广交会、厦洽会以及“中外知名企业四川行”等重大招商活动，大力推进“央企入绵”和“民企入绵”，力争引进10亿元以上项目35个，到位内资600亿元，利用外资2.5亿美元。鼓励绵阳企业和资本参与跨国并购、合作研发，建立海外生产销售基地。加大重点出口企业帮扶力度，促进外贸持续增长。

二、夯实产业基础，在构筑多点支撑上力求新突破

大力实施工业强市战略，突出工业主导地位，坚持做大增量和提升质量并举，打造绵阳经济“升级版”。

优化提升传统优势产业。重点打造千亿电子信息产业集群和五百亿汽车及零部件产业带，推动“2+4”传统优势产业持续壮大。加大技术改造创新力度，力争技改投资300亿元。加快淘汰落后产能，全面完成煤炭行业退出工作。支持长虹、九洲等龙头企业发展壮大，扶持中小微企业加快发展。认真实施“51025园区发展计划”，大力推进高新区两千亿园区、经开区千亿园区和江油工业园五百亿园区建设，支持三台、盐亭、梓潼工业园扩区调位。推动建筑业上档升级。

积极培育战略新兴产业。深入实施战略性新兴产业“2135”工程，重点发展北斗卫星导航、通用航空、新一代显示技术、3D打印、大数据和新一代互联网、新能源汽车、环保等7大新兴产业，培育30个新兴产品。积极引进中航工业，加快建设北川通航机场，壮大空管和机载电源产业。支持浪潮云计算中心发展，培育三网融合、物联网等新型业态，促进信息化建设走在全省最前列。建设中科循环经济产业园、保和富山再生资源产业园，大力发展美能科技、新亚科技等节能环保企业。

努力壮大现代服务业。以九寨旅游东环线建设为牵引，重点推介北川羌城5A级景区，建好李白故居、罗浮山、仙海等精品景区，启动涪江六峡国家级水利风景区建设，推动王朗、虎牙深度融入大九寨旅游区。全力推进总投资310亿元的科技物流产业园建设，发展第三方、第四方物流业，壮大长虹民生、安运物流等企业。大力发展电商网商、健康医疗、养老托幼、家政物业等新型业态。深入推进科技金融结合试点，着力打造金家林金融聚集区。积极培育楼宇经济，促进房地产市场稳定健康发展。

三、着力城乡统筹，在促进两化互动上力求新突破

坚持科学规划、集约建设和精细管理，因势利导、统筹推进，着力形成工农互惠、城乡一体的新型城乡关系。

加快中心城区上档升级。加大旧城改造力度，抓紧实施圣水、南河片区和朝阳厂等城中村改造。加快新区建设，完成游仙、园艺等次中心规划，拓展城市规划空间5平方公里。抓好火车站广场、警钟街等地下空间开发试点，规划建设一批立体停车位。推进乐荟城、红星美凯龙等城市综合体建设。加快西山立交接线工程、剑门路西段排水管网等治堵和排洪设施建设。全力推进涪江特大桥、绕城高速建设，确保年内建成投用。完善城管执法体制，坚决打击违法建设行为。积极发展“定制公交”，全面推广“一卡通”工程，加速建设4G网络，推动北斗新时空服务应用，推进“智慧城市”建设。

促进新型城镇健康发展。着力壮大县域经济，深入开展扩权强县试点，用好省政府支持百万人口大县改革政策。启动安县改区、三台设市工作，做好撤乡设镇和城关镇改街道办事处工作。积极推动涪城、游仙发展先进制造业和高新技术产业，支持盐亭、梓潼等丘陵地区发展传统优势产业，扶持平武、北川等民族地区发展绿色生态产业。因地制宜发展三台西平、江油青莲等商贸旅游特色镇，加快盐亭玉龙、北川曲山等小城镇百镇建设试点。赋予重点集镇相应管理权限，放开建制镇和小城镇落户限制，有序推进农业转移人口市民化，力争城镇化率46.9%。以人为核心加快公共服务均等化，完善市政基础设施，优化城镇布局形态，让城市融入大自然，让居民望得见山、看得见水、记得住乡愁。

推动农村面貌整体提升。鼓励社会资本投向农村建设，允许企业和社会组织在农村兴办各类事业，加快建设幸福美丽新村。深入实施现代农业千亿示范工程，抓好国家级水稻制种基地建设，做强国豪种业、安特药业等农业产业化龙头企业。加快江油沉水水库、梓潼凤凰水库等水源工程建设，推进农耕区“全域灌溉”。实施16.7万亩高标准农田建设，确保粮油、蔬菜等农产品安全有效供给。加大综合改革力度，加快农村土地承包经营权流转步伐。积极推广家庭经营、合作经营、企业经营等多种经营方式，新增家庭农场50家。

四、激活创新引擎，在加速科技城建设上力求新突破

认真贯彻刘延东副总理“五个新”要求，加快自主创新、推进军民融合，早日建成“五地”。

完善一套政策体系。按照“小机构、大服务”模式，努力构建科技城管理新架构。建立在绵军工单位军民融合工作推进和考核机制，着力探索军民融合创新政策，积极争创国家军民融合示范基地。借鉴中关村等自主创新示范区成功做法，用好用活部省市联动的创新服务政策，努力创建国家级创新服务体系示范基地。认真研究省政府推进科技城建设的指导意见和支持政策，完善市级政策配套。设立科技型中小微企业发展专项资金，实施“瞪羚计划”，力争新创办科技型中小企业2000户。

建设一个集中发展区。启动编制《绵阳科技城发展规划（2016-2020年）》，与科技城集中发展区规划有机衔接，形成指导科技城未来建设发展的纲领和蓝图。组建科技城集中发展区建设委员会，加强科技城大道等新区基础设施配套，重点建设欧美、台湾、民营等高新技术企业集聚区。着眼构建“一核三区多园”发展格局，集中精力建设30平方公里的“科技城军民融合创新驱动核心示范区”，支持两个国家级园区和科创区倍增跨越，推动绵阳工业园、游仙经开区、江油特钢产业园、安县工业园、北川通航产业园等园区加快发展。

搭建一批创新载体。积极推动中物院某重大专项选址绵阳，大力支持科学新城、空气动力新城、航空新城建设，打造世界一流的高精尖重大专项技术创新基地。全力争取科技部火炬中心支持，加快建设创新中心A区二期和B区、C区，用好工业技术研究院、中科育成中心、国家军民两用技术交易中心等平台。依托生产力促进中心完善大型科学仪器共享平台，新增省级企业技术中心4个。加大专利成果转化应用扶持力度，力争新增专利转化实施项目1200个、实现产值156亿元。用好人才发展专项资金，着力引进一批高层次创新创业人才特别是领军人才。

办好新一届科博会。突出“开放合作、科技创新、军民融合”主题，全力办好“2014中国科技城国际科技博览会”。抓好军民两用技术成果发布交易、高新技术产品展示展销、国际先进制造业大会、科技创新军民融合发展论坛、科技人才延揽等重点工作，打造内陆地区对外开放会展品牌。推动办会模式由政府主导向市场运作转变，努力把科博会办成国家科技成果交易和人才展示平台、国家科技信息和政策发布平台、国际科技交流合作平台。

五、狠抓消费投资，在积蓄发展后劲上力求新突破

充分发挥消费拉动增长的“稳定器”作用，超常规抓大项目上好项目，完成固定资产投资1050亿元以上。

全面激发消费潜力。认真落实国家收入分配体制改革政策，健全工资正常增长机制，提高城乡居民消费能力。大力加强绵阳农资批发市场、高水批发市场等消费基础设施建设，开展鲜活农产品“四进”工程，推进全国肉菜流通可追溯体系建设试点，积极改善消费环境。努力办好迎春购物月、国际汽车展、“绵阳造”名优特新产品展销会等活动，加快培育会展消费、租赁消费、信贷消费、信息消费等拉动力强的消费热点。

切实加快项目建设。主动对接铁路、城市基础设施、棚户区改造等国家公共投资重点方向，力争更多项目纳入国家的“笼子”、列入省上的“盘子”。积极争取奥特莱斯、苏宁生活广场等项目早日落地，尽快开工建设绵九高速和中视迪威激光显示终端等316个重大基础设施和产业项目。加快建设长虹信息化物流园二期、成绵乐客专、元通路、火车货站搬迁以及武引二期、开茂水库等433个重大续建项目，形成更大的投资额和实物工作量，确保项目顺利推进。

着力优化投资环境。持续开展“一评五制三整顿”活动，坚决打击无理阻工、扰工、揽工和违法分包、转包工程等行为，严厉惩处吃拿卡要、推诿扯皮等问题。深化“服务对象评议机关”活动，加大评议结果公开力度，努力提高政府部门办事效率。创新并联审批运行方式，认真落实领导干部联系重点项目制度，实施“一企一策”，健全完善外来企业投诉第三方处置机制。全力做好煤电油气运等生产要素配置，积极解决企业用地、融资难题，加快建设西部投资首选地。

六、提升文化实力，在打造文化强市上力求新突破

坚持社会效益和经济效益相统一，推进文化强市建设，激发文化创造活力，充分展现绵阳文化魅力。

繁荣文化产业。加快编制文化产业发展规划，积极弘扬李白文化、嫘祖文化、文昌文化，大力促进民族传统文化与现代科技文化融合发展。建设国家级文化科技融合示范基地，做优做活跃进路、越王楼和两弹城等文化产业集聚区，着力打造文化用品制造、文化创意、文化休闲等业态，发展壮大武道数码、广益游戏等文化产业企业。全力支持绵阳艺术剧院等国有院团和天青苑川剧团、爱乐合唱团等民营文艺团体发展，广泛培养文艺人才，鼓励创作一批富有绵阳特色的优秀作品。

改进文化服务。推动“三馆一站一屋”等文化场馆免费开放，支持建设综合性文化服务中心。深化基层文化志愿服务，开展“绵州讲坛”、“中华经典颂读”等文化惠民活动，办好第四届中国诗歌节。注重文化遗产保护利用，开展“非遗进校园”等活动，争取绵阳博物馆新馆年内建成开放。加强北川地震遗址保护，启动世界“双遗产”申报工作。鼓励社会力量参与公共文化服务，培育文化非盈利组织，开发利用好农村便携式影音服务系统。

加强文化管理。按照“管办分离”原则，健全文化市场监管体系，提升文化市场技术监管水平。依法打击网络谣言，净化网络文化生态，正确引导网络舆情。深入开展“净网”、“清源”、“秋风”等专项整治行动，建成20个未成年人绿色网吧，遏制违法违规经营行为。大力推进广播电视绿色频道创建活动，加大公益广告制作播出力度。深化节目创新、频道制管理体制改革，组建全媒体中心，推进传统媒体和新兴媒体融合发展。

七、发展低碳经济，在建设生态文明上力求新突破

积极争创全国生态文明建设先行示范区，探索可推广的成功经验，大力建设“美丽绵阳”。

积极推进节能减排。坚决关停不符合国家产业政策及没有治污能力的企业，对违反环保法规的新、扩、改建项目，已投产的责令停产整顿，建设中的责令停止建设。新上工业项目必须采用国内最先进技术工艺，突出循环经济导向延伸产业链，达到同行业节能先进水平。抓好重点减排项目建设，推进工业企业限期治理，强化环保硬约束，力争单位GDP能耗下降3.5%。

促进资源高效利用。健全自然资源资产管理机制，完善自然资源监管体系。建立项目用地退出机制，优化供地结构，严格执行用地投资强度标准，用好增量、盘活存量，提高资源利用效益。加快建设城北污水处理厂，逐步建成城区雨水循环利用工程，完善城市雨污水管网，提高水资源循环利用水平。加强垃圾源头治理和控制，建设城市生活垃圾焚烧发电厂，大力推进生活垃圾资源化、无害化处理。

加大生态保护力度。扎实做好国家环保模范城市复检工作，全力创建国家森林城市。重点抓好生态细胞建设，建成生态村150个、生态家园1200个。加快实施“创森八大工程”，新增绿地面积70公顷，力争森林覆盖率达到49.9%。巩固扩大平政河污染治理成果，推进芙蓉溪等11条小流域综合整治，加大饮用水源保护力度。加强秸秆禁烧和综合利用工作，做好PM2.5等环境指标监测。推行环境污染第三方治理，健全生态环境保护市场化机制。

八、保障改善民生，在增进社会和谐上力求新突破

坚持经济社会协调发展，解决好人民最关心、最直接、最现实的利益问题，努力让发展成果更多更公平惠及全市人民。

倾力办好民生实事。发挥创业基金实效，开通创业小额担保贷款绿色通道，鼓励创办中小微企业。重点做好高校毕业生、农村转移劳动力、城镇困难人员、退役军人就业，新增城镇就业4.5万人。构建和谐劳动关系，鼓舞劳动者用勤劳双手创造美好新生活。发展老年协会组织，扶持民办养老院，整合老龄学校、卫生服务站等资源，提升社区养老服务能力。健全城乡居民基本医疗保险制度，提高社会保障水平。完善住房保障体系，开工保障性住房和棚户区改造14713套、竣工9000套。加快“7·9”洪灾灾后重建，扎实做好对口援建工作。

大力发展社会事业。坚持教育优先发展、内涵发展，深化教育领域综合改革，促进城乡教育资源均衡配置。开展全民健身运动，认真备战、积极参加全省运动会。加快医药卫生体制改革，建立科学的医疗绩效评价机制，推动高新区和园艺、城南片区医疗卫生资源合理配置。创新食品药品安全监管体制，建设“食品药品安全放心市”。落实生育政策，促进人口长期均衡发展。深入实施《妇女儿童发展纲要》，维护妇女儿童合法权益。推动“国家民政工作改革创新综合观察点”建设，提高社会救助救济水平。强化统计基层基础规范化建设，扎实做好第三次全国经济普查工作。制定优秀社科成果和社科研究扶持管理办法。加强档案、慈善、红十字、残疾人、信息化、地方志等工作。

全力创新社会治理。坚持运用法治思维和法治方式化解社会矛盾，扎实推进网络理政、阳光政务、市长热线等工作。依法规范中介组织，优先发展行业协会商会类、公益慈善类、社区服务类社会组织。积极创建“法治绵阳”，健全行政执法与刑事司法衔接机制。大力建设“平安绵阳”，严厉打击各类违法犯罪活动。逗硬执行安全生产责任制，提升本质安全水平，坚决遏制重特大事故发生。完善应急管理体系，提升应急处置能力。落实党的民族宗教政策，支持民族地区均衡发展。深化国防教育和国防动员，做好民兵预备役、征兵和“双拥”工作。扎实做好文明城市测评迎检工作，提升公民素质和社会文明程度。加强人防、移民、扶贫、防震减灾、气象、水文等工作。

着力加强政府自身建设

党的十八大以来，中央把加强政府自身建设放在了更加突出的位置，以抓铁有痕、踏石留印的决心打出了改进作风的“组合拳”，为民务实清廉的政治新风扑面而来。新的一年里，我们将结合群众路线教育实践活动，把转变政府职能作为全面深化改革、推动经济发展的“马前卒”，着力向不作为、慢作为风气开刀，进一步增强政府公信力和执行力。

突出作风改进，建设服务型政府。按照“照镜子、正衣冠、洗洗澡、治治病”总要求，从源头上铲除“四风”问题滋生的土壤。着眼为民、利民、便民，采取蹲点调研、下访解难、结对帮扶等多种形式，加强政风行风建设，真正让政府服务在基层，使问题解决在基层，把公信树立在基层。大力弘扬兰辉精神，进一步增强政府工作人员服务意识、责任意识，努力建设一支信念坚定、为民服务、勤政务实、敢于担当、清正廉洁的干部队伍。

突出职能转变，建设创新型政府。积极稳妥推进新一轮地方政府职能转变和机构改革，全面落实“三个一律”要求，大力简政放权，切实承担起公共服务、市场监管、社会管理、环境保护等职责。逐步推行政府购买服务，在环卫绿化、公路养护等凡属事务性管理服务领域，原则上引入竞争机制向社会购买。树立正确政绩观，不纠结地区生产总值排位，不为了高指标而弄虚作假，优化考核指标、逗硬督导检查，推动决策落到实处。

突出依法行政，建设法治型政府。完善决策权、执行权、监督权相互协调的行政运行机制，自觉接受人大法律监督、政协民主监督和社会舆论监督，让权力在阳光下运行。建立健全重大问题专家咨询、社会公示、风险评估、合法性审查等机制程序，促进科学民主决策。切实维护城市规划的权威性和严肃性，从严加强财政预算支出执行管理。严格规范行政处罚裁量权，坚决查纠执法违法、执法寻租等行为。

突出反腐倡廉，建设廉洁型政府。坚决执行《党政机关厉行节约反对浪费条例》和《党政机关国内公务接待管理规定》，严格规范公务接待，严禁公款寄送贺年卡、明信片和购买礼物。认真落实党风廉政建设责任制，强化执法监察、效能监察、廉政监察。用好公共资源交易中心平台，进一步规范政府采购和招投标行为。加大工程建设等重点领域、关键环节专项审计力度，严肃查办违法违纪案件，全面提升政府系统廉政建设水平。

各位代表，百舸争流千帆竞发，奋勇当先时不我待！让我们紧密团结在以习近平同志为总书记的党中央周围，在省委、省政府和市委的正确领导下，坚定信心、凝聚共识，真抓实干、锐意进取，为建设西部经济文化生态强市、谱写辉煌壮丽的“中国梦”绵阳篇章而努力奋斗！
